# Common Upper Extremity Injuries in Pediatric Athletes

**DOI:** 10.1007/s12178-022-09784-1

**Published:** 2022-08-01

**Authors:** Rhonda A. Watkins, Celina De Borja, Faustine Ramirez

**Affiliations:** 1 Department of Orthopedic Surgery, Division of Pediatric Orthopedics, University of California San Francisco, San Francisco, CA USA; 2 Department of Pediatrics, University of California San Francisco, San Francisco, CA USA; 3 UCSF Benioff Children’s Hospitals, 747 52nd Street, Oakland, CA 94609 USA

**Keywords:** Pediatric sport, Elbow injuries, Shoulder injuries, Wrist injuries, Diagnostic ultrasound

## Abstract

**Purpose of Review:**

The aim of this study is to review the most recent literature on common upper extremity injuries in pediatric athletes and discuss their diagnosis, management, and outcomes. We also highlight ultrasound as a tool in their evaluation.

**Recent Findings:**

Shoulder conditions presented include little league shoulder, glenohumeral rotation deficit, acute traumatic shoulder dislocation, and multidirectional shoulder instability. Elbow conditions include capitellar OCD, medial epicondyle avulsion fracture, and medial epicondylitis. We also review scaphoid fractures and gymnast wrist. Not all physeal injuries lead to long-term growth disruption. Ultrasound has been shown to be useful in the diagnosis of scaphoid fracture, medial epicondyle avulsion fractures, and capitellar OCD. It can also be helpful in assessing risk for shoulder and elbow injuries in overhead athletes.

**Summary:**

There is a rising burden of upper extremity injuries among pediatric athletes. Knowledge of their sport specific mechanics can be helpful in diagnosis. As long-term outcome data become available for these conditions, it is clear, proper diagnosis and management are critical to preventing adverse outcomes. We highlight many of these injuries, best practice in care, and controversies in care in hopes of improving outcomes and preventing injury for pediatric athletes.

## Introduction

Pediatric athletes are at increased risk for sport-related injuries due to intrinsic factors like the impact of chronic repetitive stress on their physes and extrinsic factors such as improper sport-related technique and sport-specific kinematics [1–3]. Prior to the 2020 pandemic, there was an estimated 60 million kids aged 6 through 18 years participating in organized sport yearly [4]. Participation was steadily increasing with trends toward earlier participation in youth sport [5, 6•]. Among these sports, overhead sports like baseball and basketball have been reported as most popular. Increased sport participation has led to increased rates of sport-related injuries with a rising burden of upper extremity injuries [7–11]. This paper will review some of the common injuries seen in the shoulder, elbow, wrist, and hand of pediatric athletes with a focus on diagnosis, management and outcomes. We also highlight use of ultrasound as a diagnostic tool for these conditions.

## Little League Shoulder

Little league shoulder (LLS) is an overuse injury caused by repetitive microtrauma from torque or tractional forces on the proximal humeral physis [12–14]. Skeletally immature athletes between 11 and 16 years old (mean age 13) present with insidious onset shoulder pain associated with overhead activities, such as baseball, tennis, swimming, gymnastics, volleyball, football, or cricket. In advanced cases, symptoms may affect activities of daily living or even be present at rest. Other symptoms may include shoulder fatigue or weakness, mechanical symptoms, or secondary elbow pain [12, 13]. Physical exam reveals tenderness over the lateral aspect of the proximal humerus with decreased range of motion (ROM) or strength [12, 14]. In a retrospective study by Heyworth et al. in 2016, 30% of athletes with LLS had decreased total arc of motion from glenohumeral internal rotation deficit (GIRD) [12].

Diagnosis can be made clinically, but radiographs of the shoulder (AP view in external rotation), may reveal physeal widening, increased sclerosis or demineralization, cystic changes, metaphyseal calcification, or fragmentation. Radiographs may be normal up to 10 days from onset of symptoms and may only be remarkable after 3 weeks. Obtaining radiographs of the contralateral side may help detect subtle changes. Magnetic resonance imaging (MRI) may be used to confirm the diagnosis if radiographs are inconclusive. MRI findings consistent with LLS include physeal widening with increased signal intensity, metaphyseal bone marrow edema, adjacent periosteal edema, and subchondral cysts [7, 12, 14]. Ultrasound evaluation can be performed to assess humeral retroversion, which refers to adaptive changes that restrict physiologic derotation of the humeral head in skeletally immature athletes who participate in overhead activities. Humeral retroversion has been reported in dominant extremities of baseball players and may be associated with increased risk for shoulder or elbow injuries [12, 15, 16].

Management of LLS includes rest from overhead activities until the patient is asymptomatic, physical therapy (PT) focused on alleviating posterior capsule tightness in athletes with GIRD, periscapular and rotator cuff (RTC) strengthening, and throwing mechanics. According to Heyworth et al., the average time to full resolution and return to full competition were 2.6 months and 4.2 months, respectively [12]. Gradual return to sports and emphasis on pitching guidelines are essential in preventing recurrence [12, 14]. In the same study, recurrence of LLS was seen in 7% of athletes at an average of 7.6 months and was more likely if the athlete had GIRD [12].

Natural course for LLS is considered benign or self-limiting; however, symptoms can wax and wane and significantly affect an athlete’s ability to participate in sports. Potential complications include physeal closure, which is worrisome because the proximal physis contributes to 80% of overall humeral growth; however, these are extremely rare [12, 13]. In a 14-year retrospective study, there were no cases of physeal closure, humeral length discrepancy, or angular deformity reported as well as no differences in outcomes between athletes who had positive or negative radiographic findings, or those treated with (PT) versus those who were not [12].

## Glenohumeral Internal Rotation Deficit

Glenohumeral internal rotation deficit (GIRD) refers to adaptive changes to dominant shoulders of overhead athletes, that lead to increased external rotation (ER) and decreased internal rotation (IR), resulting in a loss of total shoulder ROM or asymmetry between dominant and nondominant shoulder. GIRD has been implicated with increased risk for shoulder and elbow injuries [12, 17–19]. However, a meta-analysis by Keller et al. in 2018 revealed that although shoulders with GIRD favored injury, it did not yield statistical significance [18].

Athletes with GIRD present with nonspecific posterior shoulder pain in the late cocking phase, shoulder stiffness, and loss of throwing velocity [19]. Diagnosis is made clinically by measuring shoulder ER and IR passively while the athlete is supine with the humerus abducted at 90° and elbow flexed at 90°. Total shoulder ROM or arc of motion is the sum of shoulder IR and ER. Athletes with GIRD may also present with scapular dyskinesia on physical exam [19, 20]. Radiographs are usually unremarkable, and in some cases may show a posterior glenoid osteophyte (Bennet’s lesion) [19]. MRI may be obtained to evaluate for concomitant pathology such as RTC (supraspinatus and infraspinatus) partial tears, bony cystic changes to posterosuperior humeral head, thickened appearance of posterior band of the inferior glenohumeral ligament (IGHL), glenoid chondral wear, and labral pathology [19].

Dynamic assessment through ultrasound may help differentiate between bony factors (humeral retroversion) versus soft tissue factors (pathologic contracture of posterior capsule) that may contribute to GIRD [20]. GIRD in the setting of an injury is managed through PT focused on improving the capsular or muscular adaptations from chronic overhead activities. Posterior capsule/RTC stretching such as the sleeper stretch and cross body stretch are associated with favorable outcomes. Arthroscopic interventions may be indicated if symptoms persist despite conservative measures [19, 20].

## Shoulder Instability

Acute traumatic shoulder instability refers to dislocation of the humeral head from the glenoid. Anterior dislocation being the most common will be the focus of this discussion. It may lead to soft tissue injuries such as avulsion of the anterior labrum and anterior inferior band of the IGHL (Bankart lesion), humeral avulsion of glenohumeral ligament (HAGL lesion), or anterior labral periosteal sleeve avulsion (ALPSA), and concomitant bony injuries including fracture of anterior inferior glenoid rim (bony Bankart lesion) with corresponding impaction fracture of the posterior superior humeral head (Hill-Sachs defect), or fractures involving the proximal humerus physis [21]. These injuries are usually sustained through a fall or a collision while the athlete’s shoulder is abducted and externally rotated [21, 22]. Previous history may include subjective feeling of instability or prior traumatic subluxation or complete dislocation events.

On exam, the athlete will present with gross deformity or shoulder asymmetry, holding the injured arm to the side with limited ROM. If the athlete presents after the joint reduction, special maneuvers such as the load and shift, hyperabduction, apprehension/relocation tests, and sulcus sign may be used to provoke symptoms or assess laxity. Radiographs of the shoulder including AP, scapular Y, and axillary views should be obtained following these injuries to assess proper joint reduction and evaluate for concomitant bony injuries. The AP view may show bony Bankart lesions with accompanying Hill-Sachs deformity or fractures of the proximal humerus, while the axillary view best visualizes concentric reduction. MRI may be utilized to evaluate the extent of soft tissue damage to the joint capsule, glenohumeral ligaments, labrum, and cartilage [21].

Multiple techniques have been described for closed reduction after anterior shoulder dislocation. Principle methods include traction-countertraction, leverage techniques, and scapular manipulation. The best technique is still up for debate. A meta-analysis revealed that traction-countertraction may provide least pain, but leverage techniques may be quickest to perform [23]. Ultimately, gentle manipulation is essential to avoid further iatrogenic injuries. Pain control is crucial while performing the procedure and sedation may be necessary for the pediatric population [21, 23]. Shoulder immobilization through an arm sling and activity modifications are recommended immediately following the injury. Proper rehabilitation includes PT focused on periscapular and RTC strengthening.

An athlete may return to sport once full ROM, strength, and ability to perform sport specific maneuvers without pain, discomfort or apprehension are achieved [21]. Recurrence of traumatic shoulder dislocations after nonoperative treatment is around 95% in individuals under 25 years old and can be as high as 100% in skeletally immature athletes. Boys between 14 and 18 years old are more likely to re-dislocate [21, 22, 24–26]. Rates for return to sport are 41% and 95% for those who are treated nonoperatively and surgically, respectively [26]. Due to concern for recurrent injuries leading to more extensive surgical management, degenerative arthropathy, and less favorable outcomes with regards to return to sport, there has been some discussion regarding earlier surgical intervention in recent literature [22, 26, 27].

Absolute surgical indications for first time anterior shoulder dislocations include open injuries, irreducible joints, or fracture/dislocation. Relative indications for first time injuries may include bony Bankart lesions with Hill-Sachs deformity, injuries sustained by overhead athletes, glenolabral articular disruption (GLAD), or ALPSA. Surgical interventions are commonly done through arthroscopic techniques and focused on joint stabilization and repairing injuries to the capsule and labrum [21]. Recurrence of anterior shoulder instability postoperatively varies depending on surgical technique. A meta-analysis revealed that re-dislocation was highest among those who underwent arthroscopic Bankart repair, in contrast to those who had open Bankart repair or Latarjet procedures [27]. Socioeconomic factors such as insurance status may also affect outcomes after acute shoulder dislocations. A retrospective review by Hung et al. revealed that publicly-insured individuals take longer to receive medical evaluation, MRI, and surgery; have higher number of previous dislocations before their initial consult; are associated with secondary bony injuries; and have higher rates of recurrent instability post-operatively [25].

Multidirectional instability (MDI) refers to anterior or posterior with inferior shoulder instability from chronic repetitive microtrauma in overhead sports [17, 21]. Athletes usually present with gradual onset subjective feeling of shoulder instability especially with overhead activities. Prior history of traumatic shoulder subluxation/dislocation may be present. Physical exam includes similar provocative maneuvers discussed in acute traumatic anterior shoulder instability. Beighton score may help assess overall ligamentous laxity [17]. Diagnosis is made clinically, although ultrasound evaluation can be utilized for dynamic assessment. A study of ultrasound findings in individuals with hypermobile type Ehlers Danlos syndrome with MDI revealed a larger subacromial area with inferior humeral head subluxation compared to the control group. Their findings suggest that symptoms from MDI may not be from impingement, and instead result from inferior humeral head displacement and loss of shoulder ROM, and should be considered when designing rehabilitation programs [28].

Management includes activity modifications and PT focused on stabilizing the shoulder through RTC and periscapular strengthening, which have shown favorable outcomes [17]. A systematic review by Longo et al revealed 60% of athletes were able return to sports at the same level while 34% returned at a lower level. Overall, only 21% of patients required surgery due to persistence of symptoms despite conservative treatment. Recurrence of MDI in those who were managed surgically were lowest among those who underwent open capsular shift (7.5%) and arthroscopic plication (7.8%) [29].

## Elbow

### Medial Epicondyle Apophysitis

Medial epicondyle apophysitis, frequently referred to as “Little League elbow,” arises in the skeletally immature athlete with open physes, typically under the age of 10. [7–10]. The developing apophysis and adjacent physis are structurally weaker than the flexor-pronator mass that originates at the medial elbow, and thus are particularly susceptible to injury due to repetitive valgus stress and traction at the medal epicondyle [8, 10, 11]. This injury occurs almost exclusively in throwing athletes such as baseball players, during the cocking phases of throwing when valgus stress on the medial elbow is maximal [8, 10, 11]. Approximately 20–50% of youth baseball players report elbow pain, and recent radiographic assessments of the medial elbow using ultrasonography and MRI have reported medial epicondyle abnormalities ranging from 10 to 50% on the throwing side [2, 3, 30•, 31–36].

Patients present with insidious onset of medial elbow pain with throwing. Physical examination reveals focal tenderness to palpation over the medial epicondyle with possible medial elbow swelling, limited elbow extension, and stiffness [8, 11, 37]. Diagnosis is clinical, but radiographs should be obtained to evaluate for widening of the medial epicondyle physis, possible fragmentation, ragged appearance, sclerosis, or trabecular thickening [7, 37, 38]. On MRI, widening of the physis can be noted with varying levels of inflammation and increased periphyseal bone marrow edema [7, 37, 38]. Several studies have examined the role of ultrasonography as a screening tool for the early detection of medial epicondyle injuries in youth baseball players and have found it effective at detecting early medial epicondyle lesions including bony cortical discontinuity or fragmentation [32, 33, 39, 40].

The management of medial epicondyle apophysitis is conservative, involving complete cessation of all throwing activities for a minimum of 4–6weeks and initiation of PT, with a focus on core, hip, and lower body strengthening and mobility [2, 15, 19]. Limited data is available on outcomes and prognosis following conservative treatment. Once full, pain-free mobility and strength have been regained and medial elbow tenderness to palpation has resolved, supervised gradual return to throwing can be initiated. This involves progression from light tosses to maximum effort pitching over 4–8 weeks [2, 15, 19]. On average, the total time to return to competitive pitching is 8–12 weeks [2, 15, 19].

### Medial Epicondyle Avulsion Fracture

Medial epicondyle avulsion fractures occur secondary to valgus stress at the elbow with increased risk as the athlete nears skeletal maturity and the apophysis begin to fuse [8, 10, 41]. It typically affects baseball pitchers ages 9–14 years [8, 10, 41]. They present acutely in an athlete with sudden pain and/or popping sensation during a single throwing motion, resulting in swelling, tenderness to palpation over the medial epicondyle, and reduced range of motion [41–43]. This injury may be associated with valgus instability and ulnar nerve symptoms [41–43]. Presence of symptoms preceding the avulsion is variable, with recent reports suggesting that the majority of athletes report pre-existing medial elbow pain prior to their acute injury [5].

Fracture displacement is often used to determine the optimal treatment. However, controversy remains about its clinical significance and the most accurate diagnostic approach to determine the degree of displacement [41, 44]. Obtaining contralateral elbow radiographs can be beneficial to distinguish between acute avulsion from anatomic variation, due to the variable age of fusion of the medial epicondyle ossification center [9, 41]. Fragment displacement is typically anterior and distal, so the displacement may be underestimated by an isolated anteroposterior radiograph [41]. Several studies have shown that internal oblique radiographs at 45° and distal humeral axial views may improve accuracy in measuring maximal displacement, as CT and MRI are typically not helpful in evaluating these injuries [41, 45–47].

Optimal management of pediatric medial epicondyle fractures continues to be an area of debate, especially with regards to surgical indications. Non-displaced or minimally displaced fractures can be treated non-operatively. Generally accepted absolute indications for operative intervention include incarcerated fragments, open fracture, and ulnar nerve entrapment [41, 42]. Relative indications include elbow instability and significant displacement. These indications are controversial however as there is no agreement regarding the definition of significant displacement (ranges from 2 to 10 mm), in addition to the aforementioned measurement controversies [41, 42, 44]. The concern about valgus instability resulting from higher rates of nonunion or malunion observed in patients treated non-operatively has led to a trend toward operative fixation of avulsion fractures in competitive upper-extremity athletes who rely on elbow stability for their sport, including baseball and gymnastics [41, 42, 44, 48].

Studies comparing operative to non-operative management demonstrate similar outcomes and ability to return to sport, although hardware removal is a frequently cited complication in the operative group and reported time to return to sports varies from 3 to 7 months [5, 43, 48–52]. In a recent study of matched operative and non-operative moderately displaced fractures in adolescent upper-extremity athletes, Axibal and colleagues reported no significant difference in the proportion of subjects who returned to the same sport (92.9% in each group), performance at pre-injury level of competition, range of motion limitations, complications, or median time to return to play [50]. However, non-operative patients tended to return to play sooner than those in the operative group (3 vs. 5.5 months, non-significant) [50].

### Osteochondritis Dissecans of The Capitellum

Osteochondritis dissecans (OCD) of the humeral capitellum results from compressive forces at the immature chondral surface of the radiocapitellar joint in the setting of excessive valgus stress or axial loading which puts the subchondral bone at risk for localized ischemia from a limited vascular supply, and altered biomechanics [8, 41, 53–55]. It occurs primarily in children and adolescents aged 10–16 years who participate in overhead throwing and axial loading activities, including baseball and gymnastics [41, 53–56]. Prevalence ranges from 1 to 4% among youth baseball players, with an increased risk in males, athletes with a longer duration of competitive play, and those who began to play at earlier ages[41–44].

Patients typically present with insidious onset, progressively worsening lateral elbow pain during activity in the dominant arm, with stiffness, loss of mobility, inability to perform at the previous level of sport, and possible mechanical symptoms or swelling [8, 53, 55, 57]. They commonly have tenderness to palpation over the radiocapitellar joint or capitellum, and may also have effusions or loss of extension, pronation, and/or supination [8, 11, 53, 55, 57]. Radiographs of the elbow in early stages may be normal, or demonstrate subtle changes including a faint subchondral lucency on the anterolateral aspect of the capitellum [7, 58, 59]. Advanced lesions may display increased lucency, sclerosis, and fragmentation of the capitellum, and possible loose bodies [7]. In addition to anteroposterior, lateral, and oblique views, anteroposterior views with the elbow in 45° of flexion should be obtained to aid in visualization, especially in radiographs with less elbow flexion or full extension among gymnasts [7, 54, 58].

Ultrasonography may be a better initial screening tool, especially for early stage lesions, and has been used extensively in the evaluation and early detection of capitellar OCD lesions, with reported positive predictive values ranging from 67 to 100% [32, 39, 53, 60–66]. Several classification systems have been proposed to classify the stability of OCD lesions, although there is limited interobserver reliability among these criteria [53, 67, 68]. MRI is helpful when initial radiographs are negative, and may reveal bone marrow edema, irregularities or fragmentation of the articular cartilage, and possible intra-articular bodies in advanced cases [7, 53]. Radiographs, MRI, CT, and ultrasonography all have a role in identifying lesions and assessing lesion instability, which is characterized by any signs of sclerosis or fragmentation and helps determine the initial management [53, 63, 66].

Stable lesions should be managed non-operatively with rest for 6 weeks, PT, cessation of overhead activities, and reassessment after 3–6 months of conservative therapy [10, 26, 39, 48]. At this time, if the patient has clinical improvement and radiographic healing, return to sport can be initiated [10, 26, 39, 48]. Good prognostic factors for non-operative treatment include early stage lesions, younger patients with open capitellar physes, smaller lesions, absence of cyst-like lesions, radiocapitellar congruity, and compliance to conservative treatment [44, 54–57]. Healing rates of 50–90% with non-operative management for early stage lesions have been reported in the literature [69–72]. In two recent studies, mean non-operative treatment duration and mean duration before returning to play were 8.3 and 6.4 months, respectively [69, 70].

Indications for operative management include failure of conservative management, unstable lesions, pain during daily activities, presence of mechanical symptoms, and/or loose bodies [63, 67]. Various surgical options exist, including retrograde drilling, internal fixation, loose body removal and microfracture, osteochondral autograft, and osteochondral allograft [53, 56]. The respective indications and differential outcomes of these various surgical techniques have been described elsewhere [53, 56, 57, 63, 73, 74]. In a recent systematic review of return to sport rates following surgical management of elbow OCD lesions, Cohen and colleagues reported a pooled rate of return to any level of sport of 98% in a mean duration of 6 months, a pooled rate of return to preinjury level of sports of 79%, and a post-operative improvement in all functional outcome scores [74]. The most common complication was revision surgery for loose body removal [74].

## Wrist and Hand

### Gymnast Wrist

Gymnast wrist is an overuse stress injury of the distal radius physis. It is most often seen in gymnasts as they bear a significant amount of their weight on their wrists at a young age leaving them subject to large compressive loads over time and susceptible to injury [75]. This area is at risk for increased susceptibility due to the proportionally greater axial loads on the distal radius (80%) compared to the ulna (20%) during weight bearing on the extended wrist [76–78]. In addition, blood flow compromise to the metaphysis and epiphysis may also be at play here [76, 79].

Athletes usually present with chronic, dull, dorsal, or radial sided wrist pain without a history of injury. Their pain is usually exacerbated by activities that load the wrist such as floor, vaulting and pommel horse. Pain at rest may be seen in advanced disease [76]. There may also be associated reports of wrist swelling. On palpation, the pain is usually localized to the distal radius physis. Pain may be worsened with hyperextension and axial loading of the wrist such as in the plank or L position on the exam table [77].

Diagnosis is made with radiographs which typically show physeal widening, metaphyseal irregularity such as beaking, and sclerosis [75]. MRI imaging is usually not needed for diagnosis but if obtained, T2 images show increased signal intensity of the physis [7]. The literature suggests that radiographic abnormalities consistent with gymnast wrist can be found in 10–85% of gymnasts with and without symptoms [78]. Treatment usually consists of cessation of weight bearing activity for 6–8 weeks. Complete immobilization in a brace or cast may be helpful for compliance.

Without treatment, athletes are at risk for progression to premature closure of the physis and a resultant discrepancy between the length of the radius and ulna [75, 80]. Clinically, this can be seen as deviation of the hand toward the radial side. The underlying ulnar positive variance that results in this case increases the risk for development of other wrist pathologies. Surgical intervention is reserved for symptomatic, partial closure of the radial physis or if there is progression to clinically unacceptable malalignment [76]. A recent retrospective analysis of immature gymnasts diagnosed with gymnast wrist found a 10% rate of growth disturbance and a 12% rate of reoccurrence [81••].

### Scaphoid Fractures

Scaphoid fractures are the most commonly encountered carpal fractures, representing about 70% of all carpal fractures [75, 76, 82]. They typically occur from a fall onto an outstretched hand, and are commonly seen in participants of contact sports such as football, rugby, or high velocity sports like skateboarding or rollerblading. In the pediatric population, these fractures have traditionally occurred most frequently at the distal pole of the scaphoid but in recent times they have been occurring with increased frequency at the waist of the scaphoid as seen in the adult population [7, 83]. This is thought to be related to increased body mass index and increased participation in high impact and extreme sports at a young age. Athletes usually present after trauma with acute wrist pain. On exam, they have tenderness to palpation of the anatomic snuffbox, scaphoid tubercle volarly, and pain with radial deviation, or pain with active wrist ROM [84].

Initial diagnostic imaging of choice is plain radiographs including scaphoid views which may or may not show a fracture line. In cases with high clinical suspicion and normal plain radiographs, advanced imaging with CT or MRI is warranted to rule out scaphoid fracture. Alternatively, repeat plain radiographs in 10–14 days may show sclerosis or early healing changes. Ultrasound imaging may be useful for sideline or point of care evaluation but has thus far shown variable sensitivity for detecting scaphoid fractures with one study reporting sensitivity of 50% [85] and another 77.8–100% [86]. This variability may be in part due to operator experience. Ultrasonography does seem to at least be reliably useful early in the disease process with a recent study showing ultrasound to be superior to radiographs in diagnosing early scaphoid fractures [87••].

Once confirmed, treatment involves immobilization in a thumb spica cast with the duration of immobilization dependent on the location of the fracture. Distal pole fractures may require 4–8 weeks of immobilization compared to up to 15 weeks for waist fractures [88]. Acute non-displaced fractures do well with casting with reports of 90% union rate [89, 90]. Surgical reduction and internal fixation with or without bone graft is the mainstay for acute displaced fractures, late presenting fractures (> 6 weeks) and chronic non unions. Reported post-surgical union rates are at least 95% [89].

## Conclusion

Rising youth sport participation and early sport specialization in popular sports like baseball, basketball, and gymnastics demand continued knowledge on the diagnosis, management, and outcomes of common upper extremity injuries seen in pediatric athletes. Furthermore, care of the pediatric athlete should involve not only special consideration of their skeletal immaturity but also of their specific sport. An understanding of sport-specific risks is essential for diagnosis and injury prevention for these young athletes. Ultrasonography is emerging as a useful tool for initial evaluation of upper extremity injuries such as scaphoid fractures, medial epicondyle avulsion fracture, and capitellar OCDs. More research is needed to solidify its role in the diagnosis of pediatric upper extremity injuries.
